# Validity of self-reported height and weight among adolescents: the importance of reporting capability

**DOI:** 10.1186/1471-2288-13-85

**Published:** 2013-06-27

**Authors:** Mette Rasmussen, Bjørn E Holstein, Ole Melkevik, Mogens Trab Damsgaard

**Affiliations:** 1 National Institute of Public Health, University of Southern Denmark, Øster Farimagsgade 5A, Copenhagen K DK-1353, Denmark; 2 Department of Child and Adolescent Mental Health, Norwegian Institute of Public Health, PO Box 4404Nydalen, N-0403, Oslo, Norway

**Keywords:** Height/weight, Self-reports, Validity, Response capability, Adolescents

## Abstract

**Background:**

This study proposes a new approach for investigating bias in self-reported data on height and weight among adolescents by studying the relevance of participants’ self-reported response capability. The objectives were 1) to estimate the prevalence of students with high and low self-reported response capability for weight and height in a self-administrated questionnaire survey among 11–15 year old Danish adolescents, 2) to estimate the proportion of missing values on self-reported height and weight in relation to capability for reporting height and weight, and 3) to investigate the extent to which adolescents’ response capability is of importance for the accuracy and precision of self-reported height and weight. Also, the study investigated the impact of students’ response capability on estimating prevalence rates of overweight.

**Methods:**

Data was collected by a school-based cross-sectional questionnaire survey among students aged 11–15 years in 13 schools in Aarhus, Denmark, response rate =89%, n = 2100. Response capability was based on students’ reports of perceived ability to report weight/height and weighing/height measuring history. Direct measures of height and weight were collected by school health nurses.

**Results:**

One third of the students had low response capability for weight and height, respectively, and every second student had low response capability for BMI. The proportion of missing values on self-reported weight and height was significantly higher among students who were not weighed and height measured recently and among students who reported low recall ability. Among both boys and girls the precision of self-reported height and weight tended to be lower than among students with low response capability. Low response capability was related to BMI (z-score) and overweight prevalence among girls. These findings were due to a larger systematic underestimation of weight among girls who were not weighed recently (−1.02 kg, p < 0.0001) and among girls with low recall ability for weight (−0.99 kg, p = 0.0024).

**Conclusion:**

This study indicates that response capability may be relevant for the accuracy of girls’ self-reported measurements of weight and height. Consequently, by integrating items on response capability in survey instruments, participants with low capability can be identified. Similar analyses based on other and less selected populations are recommended.

## Background

Body Mass Index (BMI) (kg/m^2^) is a frequently used measure for estimating weight status e.g. [1]. In large population surveys, direct measurement of height and weight is often not feasible due to restrictions in financial resources. Instead, data are commonly collected by self-reports. Self-reported data on height and weight are compromised by a number of methodological issues. Study populations of adolescents are often characterised by a substantial proportion of missing values on height and weight [2-4]. Further, weight is often under-reported [5-13] while height tends to be over-reported [5,6,8,10,12,13]. Consequently, BMI is frequently underestimated leading to misclassification as some overweight individuals are classified as being normal weight.

This paper proposes a new approach for studying bias in self-reported data about height and weight, namely to study the relevance of participants’ self-reported response capability. The relevance of considering the regularity of adolescents weighing or measuring practises and their opportunities for weighing and measuring themselves have previously been highlighted [7,14]. However, the empirical research investigating the importance of weighing oneself for the capability for responding to survey questionnaires is scarce. De Vriendt (2009) and colleagues found that Belgian adolescents who weighed themselves during the past year reported their weight with a higher accuracy than those who did not [15]. Hauck and colleagues found that a large proportion of American Indian adolescents did not know their weight or height and about half of those who reported their weight and height were uncertain about the value [16].

As indicated by the previous studies, weighing and measuring practices may be important for the ability to provide valid information on weight and height. The proximity in time between weighing and height measuring and reporting the data may be particularly important during adolescence as most adolescents experience a substantial increase in both height and weight. Also, participants’ response may be influenced by their ability to recall their height and weight. Therefore it may be useful to include items on weighing and height measuring history and perceived recall ability in survey instruments. This will allow results to be evaluated according to respondents’ capability to respond. However, the relevance of collecting such data is dependent upon the extent to which response capability for height and weight indeed is associated with the level of precision (random errors) and accuracy (systematic errors) in the self- reported measures.

The aims of this study are 1) to estimate the prevalence of low response capability for weight and height in a school-based self-administrated questionnaire survey among a population of 11 to 15 year old Danish adolescents, 2) to estimate the proportion of missing values on self-reported height and weight in relation to capability for reporting height and weight, and 3) to investigate the extent to which adolescents’ response capability influence the precision and accuracy in self-reported height and weight. Fourth and finally, the study aims to investigate the impact of students’ response capability for estimating prevalence rates of overweight.

## Methods

### Design

Data are from the Aarhus School Survey, a school-based cross-sectional questionnaire survey conducted in the city of Aarhus, the second largest city in Denmark (314.000 inhabitants). The overall aim of the study was to investigate health, health behaviour, social relations and well-being of schoolchildren in Aarhus. The survey is an interim data collection of a nationally representative survey conducted every fourth year constituting the Danish contribution to the cross-national Health Behaviour in School-aged Children (HBSC) survey [1,17]. The HBSC survey collects data from schoolchildren aged 11, 13 and 15, and the same age groups were approached in the Aarhus School Survey.

### Sampling

The Aarhus School Survey applied a strategic sampling procedure to ensure sufficient variability in socioeconomic position and ethnic background. Thirteen schools were included and all students at grade five, seven and nine were invited corresponding to the age groups of 11, 13 and 15 years. A total of 2.100 students were included in the final data file corresponding to 99% of the students present on the day of data collection and 89% of the students enrolled in the sampled classes.

### Data collection

The procedures for data collection resembled the procedures applied in the HBSC survey [1,17]. In each participating school, the school board, headmaster and students’ council had approved the study and the school nurse had been informed. The students were asked to complete the questionnaire following a standard instruction from the teacher and to return their questionnaire in sealed envelopes in order to protect their anonymity.

Parts of the internationally standardised HBSC instrument were applied for measuring socio-demographic factors, health, weight and height, health behaviours, well-being and social relations [1]. Additional items were developed for the survey including items on history of weighing and height measuring and perceived weight and height recall ability. We conducted a qualitative pilot study based on focus group discussions with students who answered the draft questionnaire. Based on the experiences from the pilot we developed the final version of the questionnaire.

### Measurements

Self-reports of weight and height were collected by the items “How much do you weigh without clothes?” (in kg.) and “How tall are you without shoes?” (in cm.).

The following questions on response capability were placed apart from the two first questions on weight and height in the questionnaire.

We obtained information on weighing and height measuring history by two items: ‘When were you last weighed/height measured? with the response categories: a) within the past week, b) within the past month, c) within the past half year, d) more than half a year ago, and e) don’t remember’. We dichotomised weighing history into being weighed ‘within the past month’ (recently) versus the combined ‘more than one month ago’ and ‘don’t remember’ categories (not recently). Height measuring history was dichotomised into being measured ‘within the past half year’ (recently) versus the combined ‘more than half a year ago’ and ‘don’t remember’ (not recently).

Perceived recall ability was measured by the following two items: ‘Many children and adolescents have trouble remembering their weight/height. How well do you remember your weight/height?’ with the following response categories: a) exactly, b) approximately, c) not very well, and c) don’t remember it. We dichotomised weight and height recall ability into ‘exactly’ and ‘approximately’ (high) versus ‘not very well’ and ‘don’t remember’ (low).

We defined two four-category combined variables on response capability for weight and height, respectively, by combining the variables on measuring history (recently/not recently) and perceived recall ability (high/low). Also, a dichotomized combined variable on BMI response capability was constructed. High BMI response capability included students who were recently weighed and height measured and who had also high recall ability for weight and height. Students not fulfilling these requirements were categorised by low BMI response capability. Students with missing data on weighing/height measuring history or recall ability were coded missing in the combined variables.

Parents’ occupational social class was measured by students’ reports of their parents’ occupation, coded into social class and categorised according to highest ranking parent into ‘high’, ‘medium’, ‘low’, and ‘unclassifiable’. Family structure was based on students’ reports on who they live with and categorised into ‘traditional family’ (two biological parents), ‘single-parent family’ (one single biological parent), ‘reconstructed family’, and missing information. Students living in other family structures were low in number (n = 15) and were left out of analyses. Further, we categorised migration status based on students’ reports on own and parents’ place of birth and students were classified into ‘Danish’, ‘immigrants’ and ‘descendants of immigrants’.

After students had completed the questionnaire survey they were invited for a consultation where direct weight and height were measured to the nearest 0.1 kg and 0.5 cm by two school health nurses at the school settings following standardised instructions. The consultations were conducted within one to three weeks following the questionnaire survey. The same weighing balance (model Seca 882) was applied for collection of all data on weight. Students were weighed wearing underclothes or the minimum clothes acceptable to them. The types of clothing were recorded. Students’ height was measured standing without shoes under standardised instructions ensuring perpendicular measures at a correctly placed height measuring scale. Following data collection, data on weights were corrected for students wearing more than underclothes (n = 860) by extracting mean weights for typical pants, skirts and long-sleeved tops. The individual extraction weight of the clothe item was done according to the student’s measured height in one of three height groups, based on the total height distribution of the sample.

Table 1 describes the distribution of variables used in analyses.

**Table 1:** Distribution of participants according to the applied variables by gender

	**Total study population**	**Boys**	**Girls**
	**(N = 2100)**	**N = 1031**	**N = 1069**
	**n (%)**	**n (%)**	**n (%)**
**Age**			
11 years	755 (36.0)	366 (35.5)	389 (36.4)
13 years	775 (36.9)	378 (36.7)	397 (37.1)
15 year	570 (27.1)	287 (27.8)	283 (26.5)
**Last weighed…**			
Within past week	707 (33.7)	315 (30.6)	392 (36.7)*
Within past month	725 (34.5)	351 (34.0)	374 (35.0)
Within past half year	354 (16.9)	199 (19.3)	155 (14.5)
Since longer	86 (4.1)	45 (4.4)	41 (3.8)
Cannot remember	200 (9.5)	105 (10.2)	95 (8.9)
Missing	28 (1.3)	16 (1.6)	12 (1.1)
**Last height measured…**			
Within past week	315 (15.0)	171 (16.6)	144 (13.5)
Within past month	634 (30.2)	304 (29.5)	330 (30.9)
Within past half year	678 (32.3)	331 (32.1)	347 (32.5)
Since longer	205 (9.8)	91 (8.8)	114 (10.7)
Cannot remember	246 (11.7)	122 (11.8)	124 (11.6)
Missing	22 (1.1)	12 (1.2)	10 (0.9)
**Can remember weight…**			
Exactly	540 (25.7)	272 (26.4)	268 (25.1)
Approximately	1147 (54.6)	556 (53.9)	591 (55.3)
Not very well	246 (11.7)	115 (11.2)	131 (12.3)
Don’t remember	133 (6.3)	72 (7.0)	61 (5.7)
Missing	34 (1.6)	16 (1.6)	18 (1.7)
**Can remember height…**			
Exactly	489 (23.3)	224 (21.7)	265 (24.8)*
Approximately	1185 (56.4)	587 (56.9)	598 (55.9)
Not very well	271 (12.9)	127 (12.3)	144 (13.5)
Don’t remember	132 (6.3)	82 (8.0)	50 (4.7)
Missing	23 (1.1)	11 (1.1)	12 (1.1)
**Response capability for weight**			
Weighed recently + high recall ability	1317 (62.7)	617 (59.8)	700 (65.5)*
Weighed recently + low recall ability	109 (5.2)	47 (4.6)	62 (5.8)
Not weighed recently + high recall ability	367 (17.5)	209 (20.3)	158 (14.8)
Not weighed recently + low recall ability	269 (12.8)	140 (13.6)	129 (12.1)
Missing	38 (1.8)	18 (1.8)	20 (1.9)
**Response capability for height**			
Measured recently + high recall ability	1420 (67.6)	698 (67.7)	772 (67.5)
Measured recently + low recall ability	203 (9.7)	107 (10.4)	96 (9.0)
Not measured recently + high recall ability	251 (12.0)	112 (10.9)	139 (13.0)
Not measured recently + low recall ability	198 (9.4)	101 (9.8)	97 (9.1)
Missing	28 (1.3)	13 (1.3)	15 (1.4)
**Response capability combined for height and weight**			
High	1023 (48.7)	491 (47.6)	532 (49.8)
Low	1026 (48.9)	517 (50.2)	509 (47.6)
Missing	51 (2.4)	23 (2.2)	28 (2.6)
**Family occupational social class**			
High	902 (43.0)	449 (43.6)	453 (42.4)*
Middle	559 (26.6)	264 (25.6)	295 (27.6)
Low	304 (14.5)	131 (12.7)	173 (16.2)
Un-classifiable	297 (14.1)	166 (16.1)	131 (12.3)
Missing	38 (1.8)	21 (2.0)	17 (1.6)
**Family structure**			
Traditional family	1160 (55.2)	585 (56.7)	575 (53.8)
Reconstructed family	191 (9.1)	80 (7.8)	111 (10.4)
Single-parent family	422 (20.1)	206 (20.0)	216 (20.2)
Other	15 (0.7)	5 (0.5)	10 (0.9)
Missing	312 (14.9)	155 (15.0)	157 (14.7)
**Migration status**			
Danish	1734 (82.6)	858 (83.2)	876 (81.9)
Decendants	236 (11.2)	104 (10.1)	132 (12.4)
Immigrants	109 (5.2)	55 (5.3)	54 (5.1)
Missing	21 (1.0)	14 (1.4)	7 (0.7)

* Significant difference in distribution between boys and girls, tested by chi square test (p < 0.05) (not accounting for the design effect caused by the applied cluster sampling). Missing values are not included in analyses.

### Ethical issues

The study complies with the Helsinki II declaration. In Denmark there is no formal agency for approval of population based surveys and the schools decide autonomously whether to participate in such surveys. The survey was conducted under full confidentiality, informed consent and voluntary participation.

### Statistical analyses

BMI was computed for each individual (BMI = kg/m^2^). The meaning of BMI-values varies depending on a child’s age and gender and BMI-values were therefore transformed into z-scores based on data tables and formulas provided by WHO [18]. Overweight was defined by z-score ≥1 according to the guidelines by WHO [18].

We used Chi-square test to test for significant differences in pair-wise comparisons of distributions. Paired-sample t-tests were conducted in order to detect significant differences in means between the direct measures and self-reported data on weight and height, respectively.

Systematic measurement error was studied by multivariate analyses of variance [19]. Here, the association between the independent variables weighing/height measure history, recall ability for weight/height, response capability for weight/height and BMI response capability and the dependent variables difference between self-reported and direct measures of weight, height and BMI z-score were analysed, respectively. Analyses were conducted in two steps: First, analyses stratified by gender and adjusted by age, family occupational social class, family structure and migration status were completed. A random effect of school was included to adjust for the design effect introduced by the applied cluster sampling approach [20]. From the literature it is evident that underestimation of weight is especially observed among girls who consider themselves to be too fat [13,21] and that overweight and obese adolescents tend to underreport their weight compared to normal weight adolescents [9,10,22-24]. The literature also documents that taller adolescents tend to underestimate their height whereas shorter adolescents tend to overestimate height [25]. These findings suggest social desirability bias in adolescents’ reports of height and weight. The concept of response capability and the applied operationalization may potentially overlap with the concept of social desirability. Therefore, secondly analyses also adjusted by measured weight and height, respectively, were conducted.

Finally, prevalence of overweight was described by BMI response capability.

Generally, since marked differences were observed between boys and girls, all analyses were therefore conducted stratified by gender. The modifying effect of gender was also tested by inclusion of an interaction term in the multivariate analyses. All statistical analyses were conducted in SAS version 9.1 (SAS Institute, Cary, NC).

## Results

Not having been height measured recently and low recall ability for height and weight were each observed among approximately one fifth of the study population while one third had not been weighed recently. The proportion of students with high response capability for weight and height was 62.7% and 67.6% respectively. The proportion of students with high capability for reporting BMI was 48.7% (Table 1).

The proportion of missing values on weight and height was significantly higher among students who had not been weighed and height measured recently and among students who reported low recall ability. Analyses of the distribution of missing values by the combined measure of response capability for weight showed that the proportion of missing values was high when students reported low recall ability irrespectively of when they were last weighed (approximately 20% compared to 5-10% in the groups of students with high recall ability). The same pattern was seen for the combined measure of response capability for height (data not shown).

Table 2 compares self-reported and direct measures of weight by weighing history, recall ability for weight and response capability for weight. Generally, significant underestimation was seen among both boys and girls (boys: -0.81 kg, SD = 4.95; girls: -1.82 kg, SD = 3.15). Analyses stratified by weighing history showed a significant underestimation of weight both among girls who are weighed recently and those who are not. The largest mean underestimation was observed among girls who are not weighed recently (−2.70 kg, SD = 4.11). Among boys, underestimation of weight was only significant among those who are weighed recently (−0.90 kg, SD = 4.24). For both boys and girls a significant underestimation of weight was observed both among students with high recall ability and students with low recall ability. Mean underestimations were larger among students with low recall ability. Analyses stratified by combined information on student weighing history and recall ability for weight showed varying patterns by gender. Among boys significant underestimation was only observed in the combination ‘being weighed recently + high recall ability’ (−0.88, SD = 4.25) and in the combination ‘not being weighed recently + low recall ability’ (−1.29, SD = 5.43). Among girls significant underestimation was observed in all four combinations. The smallest underestimation was observed among girls ‘being weighed recently + high recall ability’ (−1.42, SD = 2.39). In analyses of all operationalizations of response capability the largest standard deviation of mean difference for weight were seen among students with low response capability indicating a lower reporting precision (random measurement error).

**Table 2:** Comparisons of self-reported and directly measured weight among 11- to 15 year old adolescents by weighing history, recall ability for weight and response capability for weight

	**N**	**Weight/self-reported (kg)**		**Weight/direct (kg)**		**Difference for weight (kg)**			
		**Mean**	**SD**	**Mean**	**SD**	**Mean**	**SD**	**95**% **CI**	P value*
**Boys**	839	50.98	13.14	51.79	13.29	−0.81	4.95	−1.15; -0.81	<0.0001
**Girls**	845	47.63	10.07	49.45	10.60	−1.82	3.15	−2.03; -1.61	<0.0001
**Weighing history**									
**Boys**									
Weighed recently	572	50.74	13.12	51.64	13.30	−0.90	4.24	−1.24; -0.55	<0.0001
Not weighed recently	259	51.51	13.28	52.17	13.37	−0.67	6.27	−1.43; -0.10	0.0893
**Girls**									
Weighed recently	645	47.69	10.09	49.26	10.41	−1.57	2.64	−1.77; -1.36	<0.0001
Not weighed recently	195	47.29	10.01	49.99	11.22	−2.70	4.11	−3.28; -2.12	<0.0001
**Recall ability for weight**									
**Boys**									
High recall ability	718	50.64	13.33	51.40	13.40	−0.76	4.94	−1.12; -0.40	<0.0001
Low recall ability	112	53.13	11.95	54.34	12.58	−1.21	5.09	−2.17; -0.26	0.0131
**Girls**									
High recall ability	726	47.54	10.00	49.14	10.38	−1.61	2.83	−1.81; -1.40	<0.0001
Low recall ability	109	48.26	10.65	51.31	11.84	−3.06	4.45	−3.91; -2.21	<0.0001
**Response capability for weight**									
**Boys**									
Weighed recently + high recall ability	538	50.61	13.15	51.49	13.36	−0.88	4.25	−1.24; -0.52	<0.0001
Weighed recently + low recall ability	32	53.07	12.83	54.10	12.57	−1.03	4.21	−2.55; 0.49	0.1757
Not weighed recently + high recall ability	179	50.78	13.91	51.16	13.59	−0.39	6.62	−1.36; 0.59	0.4343
Not weighed recently + low recall ability	80	53.15	11.66	54.44	12.66	−1.29	5.43	−2.50; -0.08	0.0373
**Girls**									
Weighed recently + high recall ability	599	47.79	9.99	49.21	10.20	−1.42	2.39	−1.61; -1.23	<0.0001
Weighed recently + low recall ability	42	46.80	11.62	50.35	13.37	−3.56	4.44	−4.94; -2.17	<0.0001
Not weighed recently + high recall ability	126	46.20	9.93	48.84	11.30	−2.64	3.94	−3.33 ¸-1.94	<0.0001
Not weighed recently + low recall ability	67	49.17	9.98	51.92	10.83	−2.75	4.46	−3.84 ¸-1.66	<0.0001

* Paired *T*-test.

Table 3 compares self-reported and direct measures of height by height measuring history, recall ability for height and response capability for height. There was a significant overestimation among boys (+0.25 cm, SD = 3.47) but not among girls. For both boys and girls, analyses stratified by height measuring history showed insignificant overestimations among both students measured recently and students not measured recently. Analyses stratified by recall ability for height revealed a significant overestimation among both boys and girls with high recall ability (boys: +0.34 cm, SD = 3.21; girls: +0.19 cm, SD = 2.56). Analyses stratified by combined information on student height measuring history and recall ability for height showed significant overestimation among boys in the group ‘measured recently + high recall ability’ (+0.29 cm, SD = 3.12) and in the group ‘not being measured recently + high recall ability’ (+0.81 cm, SD = 3.54). Among girls, no significant differences were observed between self-reports and direct measures in any of the four groups. In analyses of all operationalizations of response capability the largest standard deviation of mean difference for height were seen among students with low response capability indicating a larger random measurement error.

**Table 3:** Comparisons of self-reported and direct measures of height among 11- to 15 year old adolescents by height measuring history, recall ability for height and response capability for height

	**N**	**Height/self-reported (cm)**		**Height/direct (cm)**		**Difference for height (cm)**			
		**Mean**	**SD**	**Mean**	**SD**	**Mean**	**SD**	**95**% **CI**	P value*
**Boys**	839	164.51	13.11	164.26	12.77	0.25	3.47	0.02; 0.49	0.0340
**Girls**	886	160.37	9.53	160.22	9.02	0.15	2.81	−0.04; 0.34	0.1108
**Height measuring history**									
**Boys**									
Measured recently	679	164.12	13.21	163.89	12.96	0.23	3.25	−0.02; 0.47	0.0669
Not measured recently	155	166.27	12.64	165.79	11.99	0.48	4.27	−0.20; 1.15	0.1669
**Girls**									
Measured recently	692	160.11	9.60	159.96	9.18	0.15	2.62	−0.05; 0.34	0.1427
Not measured recently	190	161.31	9.33	161.09	8.45	0.22	3.42	−0.27; 0.71	0.3713
**Recall ability for height**									
**Boys**									
High recall ability	707	164.62	13.36	164.28	13.02	0.34	3.21	0.11; 0.58	0.0046
Low recall ability	127	163.81	11.87	164.00	11.59	−0.19	4.70	−1.02; 0.63	0.6416
**Girls**									
High recall ability	746	160.39	9.59	160.20	9.21	0.19	2.56	0.01; 0.38	0.0409
Low recall ability	133	160.16	9.29	160.17	8.02	−0.01	3.91	−0.68; 0.66	0.9788
**Response capability for height**									
**Boys**									
Measured recently + high recall ability	612	164.38	13.39	164.09	13.10	0.29	3.12	0.04; 0.54	0.0225
Measured recently + low recall ability	66	161.77	11.23	162.11	11.65	−0.34	4.23	−1.38; 0.70	0.5145
Not measured recently + high recall ability	94	166.43	12.96	165.62	12.49	0.81	3.54	0.08; 1.53	0.0291
Not measured recently + low recall ability	61	166.02	12.23	166.06	11.27	−0.04	5.19	−1.37; 1.29	0.9569
**Girls**									
Measured recently + high recall ability	631	160.08	9.74	159.92	9.38	0.16	2.49	−0.03; 0.36	0.1052
Measured recently + low recall ability	60	160.50	7.97	160.42	6.90	0.08	3.66	−0.86; 1.03	0.8605
Not measured recently + high recall ability	115	162.09	8.55	161.73	8.12	0.36	2.89	−0.17; 0.89	0.1842
Not measured recently + low recall ability	73	159.87	10.30	159.96	8.88	−0.09	4.12	−1.05; 0.88	0.8607

* Paired *T*-test.

Table 4 presents mean difference in BMI z-score based on self-reported and direct measures of weight and height by BMI response capability. Significant underestimations of BMI z-scores were observed for both students with high and low BMI response capability. Especially among girls, underestimation of BMI z-scores was larger among students with low BMI response capability (−0.34 kg/m^2^, SD = 0.61) than high BMI response capability (−0.23 kg/m^2^, SD = 0.45). A larger random measurement error was observed among students with low response capability.

**Table 4:** Comparisons of z-scores based on self-reported and direct measures of weight and height among 11- to 15 year old adolescents by response

	**N**	**z-score/self-reported**		**z-score/direct**		**Difference for z-score**			
		**Mean**	**SD**	**Mean**	**SD**	**Mean**	**SD**	**95**% **CI**	P value*
**Boys**	791	−0.22	1.03	−0.08	1.01	−0.14	0.70	−0.19;- 0.09	<0.0001
**Girls**	815	−0.40	0.94	−0.12	0.95	−0.28	0.53	−0.31; -0.24	<0.0001
BMI Response capability**									
**Boys**									
High	419	−0.28	1.00	−0.11	0.99	−0.18	0.62	−0.24; -0.12	<0.0001
Low	360	−0.16	1.06	−0.04	1.06	−0.12	0.80	−0.20; -0.04	0.0053
**Girls**									
High	458	−0.40	0.95	−0.17	0.93	−0.23	0.45	−0.27; -0.19	<0.0001
Low	343	−0.39	0.93	−0.05	0.97	−0.34	0.61	−0.41; -0.28	<0.0001

* Paired *T*-test.

**High combined response capability = measured recently + high recall ability for height + weighed recently + high recall ability for weight, Low combined response capability = remaining categories.

Table 5 presents the multivariate analyses. Model 1, adjusting for age, family occupational social class, family structure and migration showed that among girls significantly larger underestimation of weight was observed among students not weighed recently (B = −1.20 kg, p > 0.0001, interaction with gender: p = 0.0015). Also among girls, low recall ability was associated with significantly larger underestimation of weight (B = −1.39 kg, p > 0.0001). Compared to girls ‘being weighed recently + having high recall ability’ all three remaining combinations of response capability for weight significantly underestimated weight (significant interaction with gender: p = 0.0033). Finally, among girls, low BMI response capability was associated with an underestimation of BMI z-score of −0.13 (p = 0.0019) (significant interaction with gender: p = 0.0105). The multivariate analyses showed no significant associations among boys. No additional significant interactions with gender were identified. In model 2, adjustment for measured height and weight were also included. Generally, a reduction in estimates was observed. Among girls the estimate for weighing history and recall ability for weight were reduced to B = −1.02 and B = −0.99, respectively. No changes in directions of associations or levels of significance were observed.

**Table 5:** Multivariate analyses of variance of associations between response capability and difference between self-reported and directly measured weight, height and BMI

	Model 1*				Model 2**			
	**Difference in self-reported and directly measured weight**				**Difference in self-reported and directly measured weight**			
	**Boys**		**Girls**		**Boys**		**Girls**	
	**B (kg)**	**P value**	**B (kg)**	**P value**	**B (kg)**	**P value**	**B (kg)**	**P value**
**Weighing history**								
Weighed recently	Ref.		Ref.		Ref.		Ref.	
Not weighed recently	0.4178	0.3306	−1.1977	<0.0001	0.5586	0.1569	−1.0197	<0.0001
**Recall ability for weight**								
High	Ref.		Ref.		Ref.		Ref.	
Low	−0.5377	0.3580	−1.3932	<0.0001	−0.2290	0.6707	−0.9902	0.0024
**Response capability for weight**								
Weighed recently + high recall ability	Ref.		Ref.		Ref.		Ref.	
Weighed recently + low recall ability	−0.1214	0.9055	−2.1313	0.0001	0.5085	0.5890	−1.6961	0.0008
Not weighed recently + high recall ability	0.7962	0.1108	−1.3159	0.0002	0.9603	0.0364	−1.2345	<0.0001
Not weighed recently + low recall ability	−0.4524	0.5145	−1.2579	0.0028	−0.2233	0.7261	−0.8671	0.0240
	**Difference in self-reported and directly measured height**				**Difference in self-reported and directly measured height**			
	Boys		Girls					
	B (cm)	P value	B (cm)	P value	B (cm)	P value	B (cm)	P value
**Height measuring history**								
Measured recently	Ref.		Ref.		Ref.		Ref.	
Not measured recently	−0.1000	0.7886	−0.0002	0.9994	−0.1867	0.5996	0.0040	0.9873
**Recall ability for height**								
High	Ref.		Ref.		Ref.		Ref.	
Low	−0.4949	0.1786	−0.1885	0.5077	−0.5693	0.1207	−0.2105	0.4483
**Response capability for height**								
Measured recently + high recall ability	Ref.		Ref.		Ref.		Ref.	
Measured recently + low recall ability	−0.6893	0.1552	0.0092	0.9824	−0.7789	0.1068	0.0282	0.9459
Not measured recently + high recall ability	−0.0610	0.8925	0.1533	0.6119	−0.1728	0.7010	0.1945	0.5183
Not measured recently + low recall ability	−0.3046	0.5553	−0.2948	0.4287	−0.4020	0.4343	−0.3449	0.3530
	**Difference in BMI z-score based of self-reported and direct measures of weight and height**				**Difference in BMI z-score based of self-reported and direct measures of weight and height**			
	Boys		Girls		Boys		Girls	
	B	P value	B	P value	B	P value	B	P value
BMI response capability***								
High	Ref.		Ref.		Ref.		Ref.	
Low	0.0404	0.4576	−0.1252	0.0019	0.0576	0.2684	−0.1042	0.0077

* adjusted for age, family occupational social class, family structure and migration. A random effect of school was included to adjust for the design effect introduced by the applied cluster sampling approach.

** adjusted for age, family occupational social class, family structure, migration and direct measure of height and/or weight. A random effect of school was included to adjust for the design effect introduced by the applied cluster sampling approach.

***High BMI response capability = measured recently + high recall ability for height + weighed recently + high recall ability for weight, Low combined response capability = remaining categories.

Table 6 presents overweight prevalence based on self-reports and direct measures by BMI response capability. Among boys, the difference in absolute underestimation of overweight prevalence between students with low and high BMI response capability was 0.58 percentage points being highest among boys with high response capability. Among girls, the difference constituted 1.33 percentage points with the underestimation being largest among girls with low response capability. Generally, the overweight prevalence was higher among students not measured recently compared to those measured recently and among student with low recall ability compared to students with high recall ability (data not shown). This is reflected in table 6 showing that the overweight prevalence was highest among students with low BMI response capability.

**Table 6:** Overweight prevalence based on self-reported and direct measures of weight and height by BMI response capability

	**Boys**			**Girls**		
	**% overweight/self-reported**	**% overweight/direct measures**	**Absolute difference**	**% overweight/self-reported**	**% overweight/direct measures**	**Absolute difference**
	**10.97**	**15.64**	**4.67**	**7.06**	**13.17**	**6.64**
BMI response capability*						
High	9.55	14.48	4.93	6.91	12.40	5.49
Low	12.71	17.06	4.35	7.27	14.09	6.82

*High combined response capability = measured recently + high recall ability for height + weighed recently + high recall ability for weight, Low combined response capability = remaining categories.

## Discussion

The presented results from a Danish population of school children aged 11 to 15 showed that approximately one third of the students have low response capability for weight and height, respectively. Every second participant had low response capability for BMI. Students who reported low recall ability were less likely to report their weight in the survey irrespective of when they were last weighed. The same pattern was found for response capability for height. This indicates that reporting of weight and height depend more on recall ability than on weighing and height measuring history.

Both boys and girls underestimated their weight. The average underestimation was relatively small, 0.8 kg for boys and 1.8 kg for girls. This difference by gender is in line with a number of previous studies [6,8,9,21,25-27] while other studies find no differences between boys’ and girls’ reports [5,7,12,14,23,28]. Only among girls, a significant larger systematic underestimation of weight was seen among those who are not weighed recently. This result is in line with the findings of the previous Belgian study by De Vriendt (2009) [15]. Significantly larger underestimation was also seen among girls who do not recall their weight. When analysing the combined measure for response capability for weight having ‘weighed recently + high recall ability’ as the reference group all remaining combinations of weighing history and recall ability show a significantly larger underestimation of weight. While no systematic under- or over-reporting of weight by response capability was detected among boys, both among boys and girls the results indicate a larger reporting error (random measurement error) among students with low response capability.

Generally, adolescents tend to overestimate their height [5,6,8,10-12,28] and in the present study this is observed among boys. A few studies show overestimation of height especially among girls [13-15,27]. It is however questionable whether the difference observed in the present study is practically relevant as it does not exceed the precision of the height measures. There was no significant difference between mean self-reported height and mean direct measures of height among girls. The multivariate analyses showed that for both boys and girls neither height measuring history, recall ability for height or response capability for height are systematically related to the difference in self-reported and directly measured height. While no systematic difference in misclassification of height by response capability was detected, both among boys and girls the results indicate a larger random measurement error among students with low response capability.

BMI z-scores were underestimated when based on self-reports of weight and height irrespective of gender and BMI response capability. A gender difference was identified as girls with low BMI response capability systematically underestimated their BMI z-scores more than girls with high BMI response capability. Difference in BMI z-scores among boys did not vary across BMI response capability. These differences by gender are also reflected in the analyses of overweight prevalence. Among boys, the difference in underestimation of overweight prevalence constituted only 0.58 percentage points (with the largest underestimation among boys with high response capability) while the difference constituted 1.33 percentage points among girls when comparing students with low and high BMI response capability. Generally, the overall misclassification of height and weight from self-reported data resulted in an underestimation of the proportion of overweight boys of approximately 5% and an underestimation of overweight girls of approximately 6%.

Among both boys and girls low response capability seems to be consistently associated with a larger random measurement error while a systematic underestimation of BMI z-score and overweight prevalence due to low response capability was only observed among girls. These finding were due to a systematic underestimation of weight among girls who were not weighed recently and among girls with low recall ability for weight. The results therefore indicate that integrating measures of response capability for weight and height among adolescents in questionnaire surveys may be appropriate for identifying adolescent girls with an increased risk of reporting erroneous information on weight. Following, analyses and conclusions drawn based on self-reported data only can be evaluated and adjusted accordingly, e.g. by comparing analyses conducted with and without inclusion of adolescents with low response capability. One way to benefit from information about response capability is to carry out sub-group analyses among participants with high response capability. If analyses in such sub-groups produce prevalence levels and associations which are very different from analyses on the entire study population, this would be an indication of severe problems of misclassification in the entire study population. The present study is however the first of its kind and additional studies in other and less selected populations are needed to generate a more general picture on the influence of response capability for reporting height and weight among adolescent boys and girls. Generally, it should however be prioritized that possible adaptions of study designs are conducted to minimise the proportion of students with low capability for reporting height and weight. One approach could be to encourage participants to weigh and measure themselves prior to data collection. This has been suggested earlier by Wang et al. (2002) [7].

In the presented multivariate analyses measured height and weight were included in the final models. This led to some reduction in estimates indicating that some overlap may exist between the applied measure of response capability and social desirability when adolescents report weight and height. This finding is supported by the fact that overweight prevalence is higher in the groups of students who report not having been measured recently compared to those who are and in the groups of student with low recall ability compared to students with high recall ability. Still, despite adjusting for measured values significantly larger systematic underestimations were seen among girls with low response capability compared to girls with high response capability.

The presented results should be evaluated in light of the methodological approach employed. For the concept of response capability a number of assumptions are made. We define response capability by time since last weighing/height measure and ability to recall. This approach does not consider other factors including availability and accuracy of home equipment for weighing and measuring, how the weighing and measuring are conducted, and whether the child is aware of the measured values. The participation rate was generally high and we do not anticipate substantial selection bias. However, the study is not representative and the prevalence figures cannot be generalised across populations. We propose repetition of this study in other and less selected study populations.

## Conclusion

The present study showed that one third of students aged 11 to 15 years had low response capability for height and weight when responding in a self-administrated questionnaire survey. Both boys and girls underestimate their weight. Also among both boys and girls the random measurement error tended to be largest among students with low response capability while only among girls with low response capability there was a systematically larger underestimation of weight. Consequently, a similar larger underestimation of BMI z-score and overweight prevalence was found among girls with low response capability. Boys over-reported their height, and for both boys and girls the random measurement error tended to be larger among students with low response capability. For both boys and girls, there was no systematic difference in reporting height by response capability. The present study indicates that this approach may be particularly relevant for studies including self-reported measurements from girls. Repetition of this study in other and less selected study populations is recommended.

## Competing interests

The study was financed by the Nordea Foundation. The Nordea Foundation was not involved in the study design, data analyses or interpretation of results. The authors declare no competing interests.

## Authors’ contributions

MR is the principal investigator of the Aarhus School Survey. MR conceived and coordinated the study, contributed to its design, acquisition of data, statistical analyses, interpretation of data and drafted the manuscript. BH co-conceived the study and contributed to its design and coordination and revised the manuscript critically. MTD contributed to the design of the study and to the statistical analyses and revised the manuscript critically. OM contributed to the design of the study, interpretation of data and revised the manuscript critically. All authors read and approved the submitted manuscripts.

## Pre-publication history

The pre-publication history for this paper can be accessed here:

http://www.biomedcentral.com/1471-2288/13/85/prepub
